# Using Radiofrequency Echographic Multi-spectrometry in the Follow-Up of Patients With Axial Spondyloarthritis and Psoriatic Arthritis

**DOI:** 10.7759/cureus.84370

**Published:** 2025-05-18

**Authors:** Ionut-Andrei Badea, Mihai Bojinca, Violeta Bojinca, Andreea-Ruxandra Ilina, Madalina-Stefania Vulcan, Stefan-Sorin Arama

**Affiliations:** 1 Department of Internal Medicine and Rheumatology, Dr. I. Cantacuzino Clinical Hospital, University of Medicine and Pharmacy "Carol Davila" (UMPCD), Bucharest, ROU; 2 Department of Rheumatology and Internal Medicine, Sfanta Maria Clinical Hospital, University of Medicine and Pharmacy "Carol Davila" (UMPCD), Bucharest, ROU; 3 Department of Internal Medicine, Colentina Clinical Hospital, Bucharest, ROU; 4 Department of Rheumatology, Colentina Clinical Hospital, Bucharest, ROU; 5 Deparment of Physiopathology, University of Medicine and Pharmacy "Carol Davila" (UMPCD), Bucharest, ROU

**Keywords:** axial spondyloarthritis, bone mineral density, fragility score, psoriatic arthritis, radiofrequency echographic multi-spectrometry

## Abstract

Introduction: Spondyloarthritis comprises a group of diseases with similar clinical, paraclinical, and imagistic findings. Another common element in this group is its association with osteoporosis and increased bone fragility, which can have dire consequences on the quality of life of these patients.

Materials and methods: A prospective study was performed in order to evaluate the bone density and fragility parameters offered by radiofrequency echographic multi-spectrometry (REMS). A group of 70 patients, 49 with axial spondyloarthritis and 21 with psoriatic arthritis, were evaluated using REMS and followed up at one year to see if there is any modification of bone mineral density (BMD), T scores, and of the fragility score (FS) under treatment.

Results: Both groups showed slightly improved values for BMD and T-scores, with significant improvement for BMD in both groups with p<0.001, with an maximum increase of 0.03 g/cm^2^ in the lumbar site and 0.02 g/cm^2^ in the femoral site, while no statistical significance was observed for T-scores, p=0.619 for lumbar spine and p=0.93 for the femoral examinations under biological therapy and combined conventional and biological treatments. A more impressive outcome was seen in the same treatment group regarding the improvement of the FS in these groups (with a mean score reduction of 11.11, p<0.001).

Conclusion: REMS is a method that need to be further studied in axial spondyloarthritis and psoriatic arthritis in order to describe it's usefulness in these specific pathologies. FS can be a useful tool in assessing bone health under different treatment conditions. Although improvement was seen in all markers under biological treatment and combined treatment, the most favourable results were observed in patients under biological disease-modifying antirheumatic drug (bDMARD) and combined conventional synthetic disease-modifying anti-rheumatic drug (csDMARD) and bDMARD therapies.

## Introduction

Osteoporosis and bone fragility are common complications observed in the spondyloarthritis (SpA) group [1]. A decrease in bone density has been documented in the early stages of SpA, due to mechanisms different from those seen in rheumatoid arthritis (RA) or other inflammatory rheumatic diseases [1]. While in RA, the increased risk of osteoporosis is associated with high cytokine activity (especially in seropositive forms of the disease) and with the appearance of marginal erosions directly linked to inflammatory activity at the synovial level [2], in SpA, the dominant features are osteoformative, such as juxta-articular osteosclerosis, formation of syndesmophytes, and the presence of enthesophytes [3].

The argument that fragility arises merely due to decreased joint mobility, i.e., ankylosis itself, is insufficient, since demineralization has been demonstrated in several studies to precede structural lesions [4]. Additionally, systemic corticosteroid therapy is rarely used in SpA, compared to other inflammatory joint diseases (RA, lupus erythematosus, etc.), and clinicians also have the option of local corticoid therapy [5]. There are also differences between the various diseases within the SpA group. While in axial spondyloarthritis (axSpA), the prevalence of osteoporosis ranges between 11.7% and 34.4% [6], in psoriatic arthritis (PsA), Gulati et al. found similar rates between patients and the general population [7].

The pathogenic mechanisms behind decreased bone density in SpA are not fully described. The abundance of proinflammatory cytokines may explain the onset of bone fragility [8]. However, the main cytokines involved in the pathogenesis of SpA are tumor necrosis factor alpha (TNFα), interleukin (IL)-17, IL-12, and IL-23 [9]. For TNFα and IL-17, there is clear evidence of interaction with bone precursor cells, promoting osteoclastogenesis and activation of bone resorptive mechanisms [6,10]. In murine experiments, IL-12 was more commonly associated with bone loss, while IL-23 had a regulatory role on bone turnover and bone formation [11]. It's important to mention that IL-17 also has an osteoformative effect, leading to the formation of new bone, specific to SpA [6].

Disease-modifying antirheumatic drugs (DMARDs) are generally immunosuppressive or immunomodulatory treatments that allow for adequate control of chronic inflammatory rheumatic diseases. They are mainly classified based on the spectrum and degree of activation of pathogenic cytokines or the cells involved in the pathological inflammatory or immune response [12]. In the case of SpA, three classes of DMARD therapies are currently described, each encompassing a wide range of molecules that are either approved, pending approval, or under phase II or III clinical trials. The three classes are represented by conventional synthetic DMARDs (csDMARDs), which have a nonspecific immunosuppressive role, acting at the level of immune and inflammatory cells to reduce cytokine production. Three are mainly used in clinical practice: methotrexate, leflunomide, and sulfasalazine [13]. The more targeted therapies are represented by biological DMARDs (bDMARDs), molecules produced through genetic engineering and cellular manipulation to create monoclonal antibodies, soluble cytokine receptors, or receptor-blocking fusion proteins. These medications have a high affinity for blocking key cytokines and cells involved in the pathogenic process of SpA and inflammatory rheumatic diseases in general [9].

Eleven such therapies are approved for use in SpA, classified based on their targeted mechanism [13]: TNFα blockers (adalimumab, infliximab, certolizumab pegol, golimumab, and etanercept), IL-17/17A blockers (secukinumab and ixekizumab), IL-12/23 blockers (ustekinumab), IL-23/23A blockers (guselkumab and risankizumab), and selective co-stimulation modulators (sometimes refered to as T-cell activation blockers in clinical practice (anti-CD80/86: abatacept) [13]. Lastly, targeted synthetic DMARDs (tsDMARDs), represented by Janus kinase inhibitors (JAK inhibitors), which act by blocking intracellular communication pathways through the inhibition of the JAK-STAT pathway, are one of the latest additions to the therapeutic spectrum for these pathologies and are represented by tofacitinib, baricitinib, upadacitinib [13].

These therapies are effective in controlling clinical manifestations, inflammation, and imaging progression associated with different forms of SpA, including PsA [14]. Moreover, most have proven useful in controlling complications and extra-articular manifestations within this pathological group [15]. Their effect on bone mineralization remains unclear at this time. Results found in scientific literature are either contradictory, or the studies highlighting a positive effect are influenced by various confounding factors [16,17].

While the assessment of bone mineral density (BMD), fragility, and microarchitectural changes is based on multiple methods, the standard for clinical assessment remains dual-energy X-ray absorptiometry (DXA) [18]. The clinical applicability of other evaluation methods (e.g., quantitative ultrasound (QUS), quantitative computed tomography (QCT), magnetic resonance imaging (MRI)) has not yet been proven, partly due to the high costs involved or the inability to assess the axial skeleton [19]. The advantages of DXA, namely accessibility, cost, and speed, can be overshadowed in SpA by the risk of obtaining overestimated BMD values, particularly in individuals with vertebral structural changes [20]. Overestimation of BMD has also been identified in other clinical scenarios, such as chronic spinal cord injuries [21], spondylosis [22], and diffuse idiopathic skeletal hyperostosis (DISH) [23]. Another method, which has the same accuracy as DXA, is radiofrequency echographic multi-spectrometry (REMS) (Echolight S.p.a., Viale Cipro, Lecce, Italy), which has shown high accuracy in diagnosing osteoporosis [24]. 

In this study, we tried to evaluate the bone density and fragility parameters offered by REMS. Our main objective was to test whether the medication usually prescribed by rheumatology physicians for axSpA and PsA had any effect on bone mineralization and microarchitectural markers. Secondly, we wanted to see if REMS can offer relatively reliable results for BMD, T-score, and FS by checking the correlations between these values in lumbar and femoral examinations.

## Materials and methods

A prospective study was conducted at Dr. I. Cantacuzino Clinical Hospital, Bucharest, Romania, from January 2020 to February 2023. Ethics Committee approval was obtained from the Dr. I. Cantacuzino Clinical Hospital. Patients were informed about the purpose of the study and the procedures involved, and all signed an informed consent form. Recruitment was carried out in the aforementioned public hospital and a private clinic, Novalife Clinic, in Bucharest, while the densitometric evaluation was performed at another private clinic, Osteodensys Clinic, in Bucharest. Patients visiting the hospital for routine check-ups between January 2020 and January 2022 were screened. We looked for patients who were diagnosed with either axSpA or PsA.

Inclusion and exclusion criteria

Fulfillement of the Assessment of SpondyloArthritis international Society (ASAS) classification criteria for axSpA [25] and Classification Criteria for Psoriatic Arthritis (CASPAR) criteria for PsA, respectively [26], was essential in enrolling the patients. Exclusion criteria were the presence of other comorbidities that are known to affect bone metabolism, such as hypothyroidism, uncontrolled diabetes mellitus, concomitant medication with oral or intramuscular corticosteroid treatment, or recent history of oral or intramuscular corticosteroid treatment, as well as any concomitant antidepressant therapies. 

A total of 70 patients diagnosed with axSpA and PsA were finally enrolled in the study.

Procedure

Patients fulfilling the eligibility criteria were enrolled and underwent anamnesis and clinical examination. Relevant medical documents (including recent lab results or results obtained during the current routine hospital visits) were obtained. Medical and medication history were taken, weight and height were measured, and blood tests were performed (normally recommended during routine hospital visits or through referral-based laboratory testing, with no additional costs incurred by the patients). After this, a bone densitometric examination was carried out using REMS in order to obtain the values of BMD, T-score, and FS. 

After one year, the patients arrived for the second visit, where the same tests were performed. Of interest was the presence or absence of any systemic inflammatory syndrome, objectified by elevated C-reactive protein (CRP) levels, which was evaluated during both visits. All clinical examinations and REMS analysis were performed by one investigator, while blood tests were done during routine visits, thus at the same laboratory, in a standardized manner. 

Data analysis

The data (demographics, CRP values, BMD, T-score, FS, weight and height measurements, as well as medical history, treatment history and current specific treatment for axSpA and PsA) were stored electronically in the patient's file and in Microsoft Excel worksheets (Microsoft Corporation, Redmond, Washington, United States), and statistical analysis was performed afterwards, by using DATAtab Online Statistics Calculator (DATAtab e.U. Graz, Austria; https://datatab.net). The aim was to test the correlation between measurements with Pearson correlation, and repeated measures ANOVA was applied to check the statistical significance of the data acquired.

## Results

The final study group consisted of 70 patients, of whom 49 were diagnosed with axSpA (42 male and seven female patients, with a mean age of 39.4 years) and 21 were diagnosed with PsA (13 female and eight male patients with a mean age of 42.7 years). Main treatment characteristics and the presence of inflammation can be seen in Table 1.

**Table 1:** Frequency of inflamation depending on treatment (N=70) bDMARD: biological disease-modifying antirheumatic drug; NSAID: non-steroidal anti-inflammatory drug; csDMARD: conventional synthetic disease-modifying antirheumatic drug

Treatment Type	No Inflammation, n (%)	Inflammation, n (%)	Total, n (%)	Chi²	df	p value
bDMARD	13 (18.57%)	1 (1.43%)	14 (20%)	2.58	1	0.091
NSAIDs	6 (8.57%)	8 (11.43%)	14 (20%)	1.47	1	0.225
csDMARD	15 (21.43%)	0 (0%)	15 (21.43%)	5.87	1	0.015
None	8 (11.43%)	4 (5.71%)	12 (17.14%)	-	-	-
csDMARD + bDMARD	14 (20%)	1 (1.43%)	15 (21.43%)	3.14	1	0.076
Total	56 (80%)	14 (20%)	70 (100%)	20.27	4	<0.001

**Figure 1:**
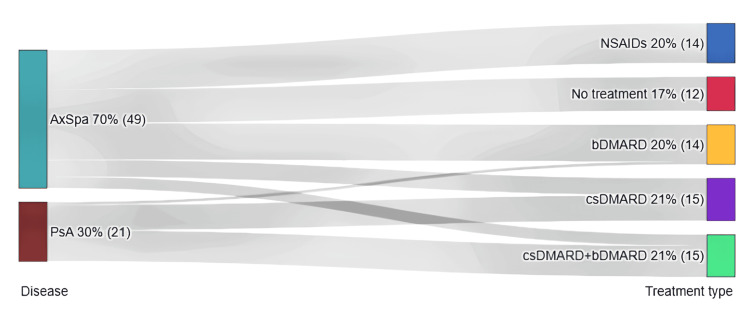
Sankey diagram showing the treatment followed in the two studied groups dark green: AxSpA group; dark red: PsA group; blue: only NSAID treatment; red: no treatment; yellow: only bDMARD treatment; purple: only csDMARD treatment; light green: combined csDMARD+bDMARD treatment only. AxSpA: axial spondyloarthritis; PsA: psoriatic arthritis; NSAID: nonsteroidal anti-inflammatory drug; bDMARD: biological disease-modifying antirheumatic drug; csDMARD: conventional synthetic disease-modifying antirheumatic drug

A Sankey diagram was made in order to show the type of treatment followed in the two study groups (Figure 1). Table 2 summarizes the treatment options and the percentage of individuals in each pathological group. A clear difference is observed between singular bMARD therapy, which is more frequent in axSpA than in PsA. In the case of PsA patients, the combination of csDMARDs and bDMARDs was preferred.

**Table 2:** Treatment type in study groups AxSpA: axial spondyloarthritis; PsA: psoriatic arthritis; NSAID: nonsteroidal anti-inflammatory drug; bDMARD: biological disease-modifying antirheumatic drug; csDMARD: conventional synthetic disease-modifying antirheumatic drug

Disease	bDMARD, n (%)	NSAIDs, n (%)	csDMARD, n (%)	No treatment, n (%)	csDMARD+bDMARD, n (%)	Total, n (%)	Chi^2^	df	p value
AxSpa	13 (18.57%)	14 (20%)	6 (8.57%)	12 (17.14%)	4 (5.71%)	49 (70%)	-	-	-
PsA	1 (1.43%)	0 (0%)	9 (12.86%)	0 (0%)	11 (15.71%)	21 (30%)	-	-	-
Total	14 (20%)	14 (20%)	15 (21.43%)	12 (17.14%)	15 (21.43%)	70 (100%)	34.47	4	<0.001

**Figure 2:**
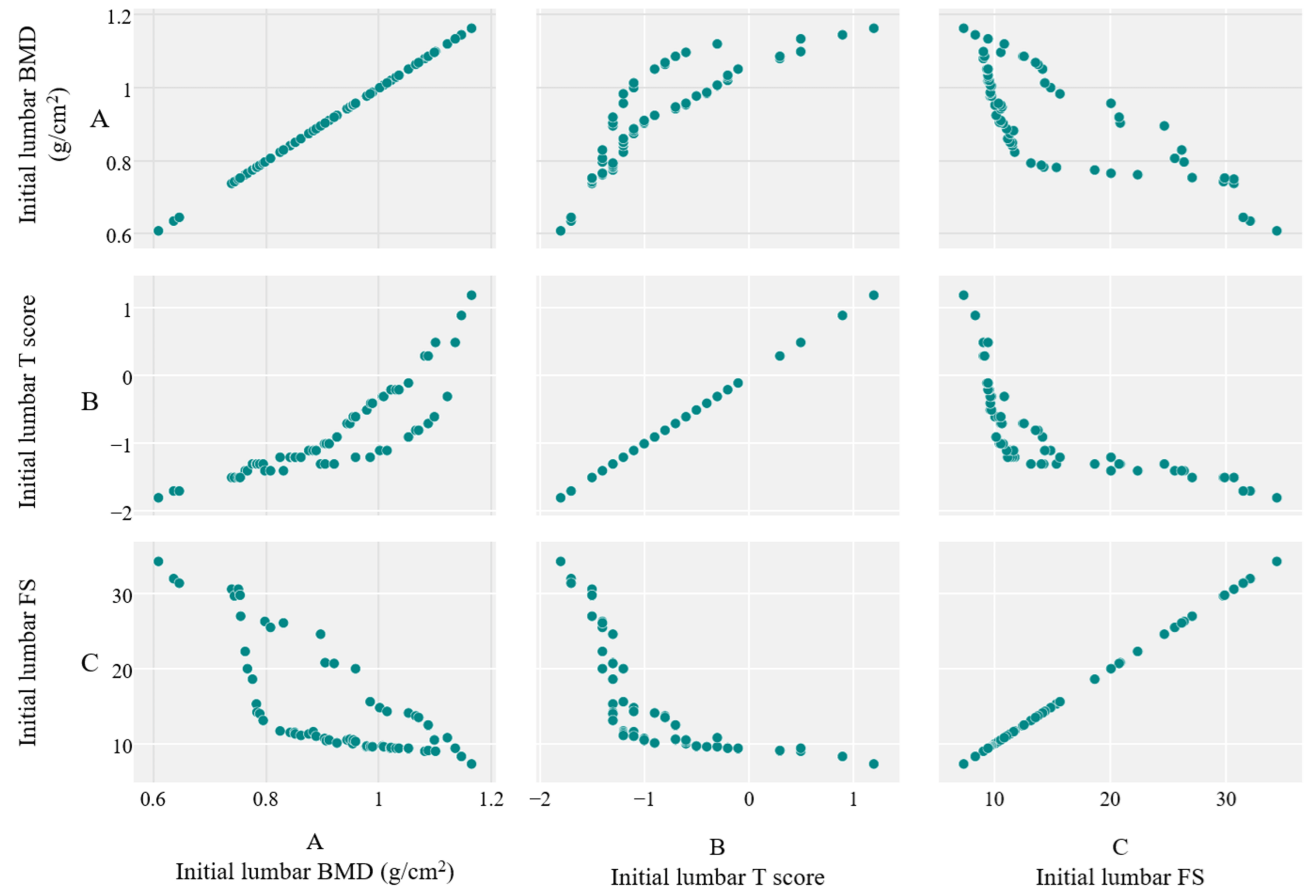
Scatter diagrams showing the correlation between initial values of BMD (A), T score (B), and FS (C) in the lumbar spine. BMD: bone mineral density; FS: fragility score

A high positive correlation obtained through the Pearson test was observed between measurements of BMD and T score, both in the lumbar spine (r=0.86, p<0.001), with a high negative correlation between BMD and FS in the same evaluation site (r=-0.78, p<0.001) (Figure 2). These suggest that REMS might offer valid results, without a high level of randomness in the measurements.

**Figure 3:**
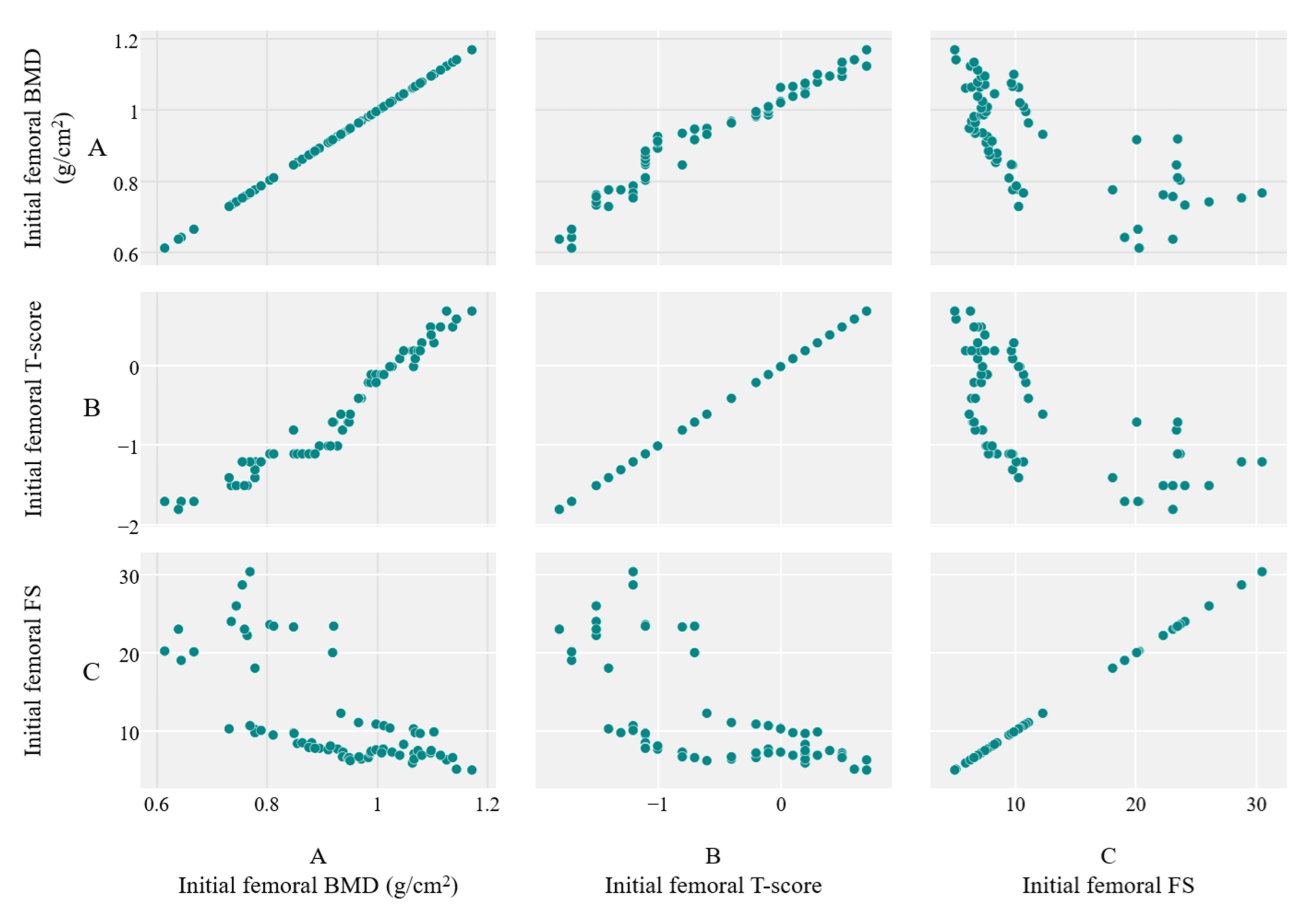
Scatter diagrams revealing the correlations between measurements of initial BMD (A), T score (B), and FS (C) of the femoral site. BMD: bone mineral density; FS: fragility score

The same type of observations can be seen when examining the femoral site. Applying the Pearson correlation test yielded a highly positive correlation between BMD and T score (r=0.97, p<0.001) and a negative correlation between BMD and FS, respectively (r=-0.6, p<0.001). This data suggests that FS is truly independent of BMD values and could be an accurate marker of bone fragility through microarchitectural changes of the bone. A graphical view of these correlations in the femoral site can be consulted in Figure 3.

Further, we followed up on the evolution of BMD measurements, along with T scores and FS scores for both skeletal sites. The analysis was performed in time in the whole study group and separately in axSpA and PsA. ANOVA analysis showed there was a significant evolution in time of BMD (p<0.001), T scores (p=0.46), and FS (p<0.001) of the lumbar spine in relationship with the treatment given in all participants (95%CI=-0.01, 0). When examining separately, axSpA patients also showed a significant increase of lumbar BMD under treatment (p=0.001; on Bonferroni test, probably false negative values for 95%CI=-0.01, 0 were obtained judging by the evolution observed for BMD), with most improvements seen in patients taking bDMARDs and combined therapies (csDMARDs and bDMARDs, 95%CI=-0.04, -0.02 on Bonferroni test). The same cannot be said about lumbar T-score variation (p=0.672), which was not influenced in any way by treatment. Reduction of FS was also noticed after one year of treatment with bDMARDs and combined therapies (p<0.001).

**Figure 4:**
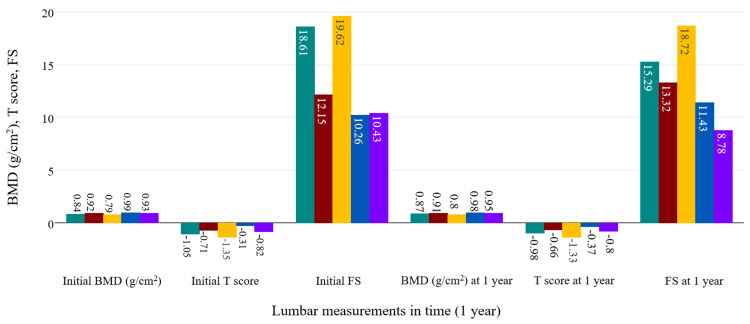
Evolution of the lumbar measured parameters under treatment in the axSpA group. green: bDMARD; red: NSAIDs; yellow: csDMARD; blue: no treatment; purple: csDMARD+bDMARD bDMARD: biological disease-modifying antirheumatic drug; NSAID: non-steroidal anti-inflammatory drug; csDMARD: conventional synthetic disease-modifying antirheumatic drug; axSpA: axial spondyloarthritis

**Figure 5:**
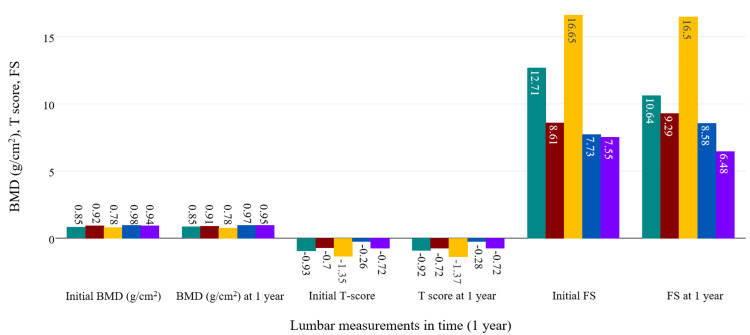
Evolution of femoral measurements in axSpA group. green: bDMARD; red: NSAIDs; yellow: csDMARD; blue: no treatment; purple: csDMARD+bDMARD bDMARD: biological disease-modifying antirheumatic drug; NSAID: non-steroidal anti-inflammatory drug; csDMARD: conventional synthetic disease-modifying antirheumatic drug; axSpA: axial spondyloarthritis

**Figure 6:**
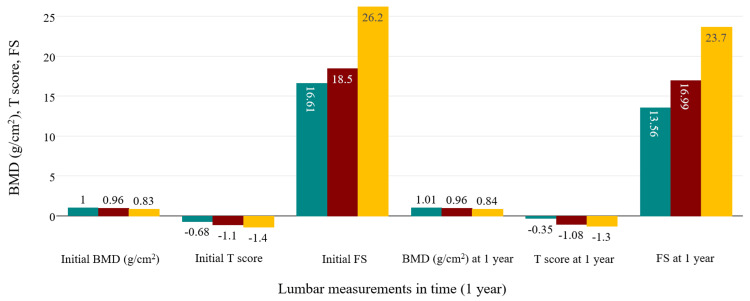
Evolution of lumbar parameters in the PsA group. green: csDMARD+bDMARD; red: cDMARD; yellow: bDMARD csDMARD: conventional synthetic disease-modifying antirheumatic drug; bDMARD: biological disease-modifying antirheumatic drug; PsA: psoriatic arthritis

**Figure 7:**
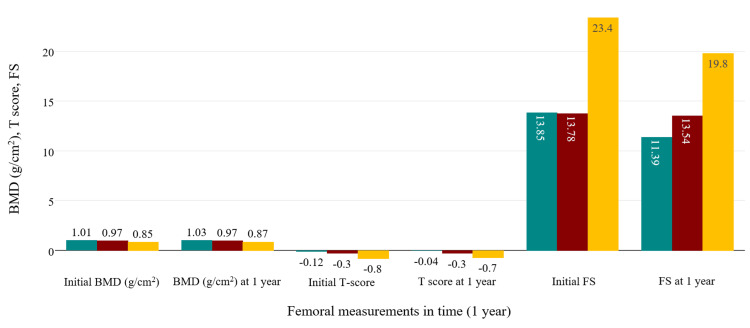
Evolution of femoral measurements in the PsA group. green: csDMARD+bDMARD; red: cDMARD; yellow: bDMARD csDMARD: conventional synthetic disease-modifying antirheumatic drug; bDMARD: biological disease-modifying antirheumatic drug; PsA: psoriatic arthritis

ANOVA analysis for the PsA group was similar to the previous examinations in regards to statistical significance, showing an increase of BMD (p<0.001) and a reduction of FS (p<0.001) under bDMARD and combined therapies. T scores were positively influenced, but statistical testing didn't show strong significance (p=0.124). Figure 4 and Figure 5 summarize all the modifications observed in the axSpA group, while Figure 6 and Figure 7 contain the evolution of the measured parameters of the PsA group.

There is a statistically significant impact on BMD and FS in relation to treatment in both study groups (p<0.005), but with no clear significant level obtained regarding T score variation on the lumbar spine (p=0.672 for axSpA and p=0.124 for PsA).

## Discussion

The usefulness of REMS in monitoring the evolution of patients over time, under various therapies, is the central element of the study, with FS proving to be an extremely valuable tool for defining fracture risk based on bone fragility [27]. Various therapies currently used in patients with axSpA and PsA have been evaluated regarding their effect on bone quality [28,29]. No study has been found that used REMS as a method of following up patients under specific DMARD treatment. It is clear that DMARD therapies should not be used specifically for controlling bone mineralization abnormalities arising in SpA group diseases [30]. However, they may have a beneficial effect on bone density and fragility, acting more as an "adjuvant" treatment alongside targeted therapies that specifically address bone mineralization in this context. This role stems from their local and systemic anti-inflammatory effects, targeted at disease pathogenesis, and to a lesser extent from their influence on bone microarchitecture and bone metabolic markers [31,32]. Systemic and localized inflammation has a negative impact on bone mineral density, inducing microstructural changes and significantly increasing fracture risk. This observation is generally valid across all types of inflammatory rheumatic diseases, including axSpA and PsA [6].

AxSpA is much more frequently associated with lower bone density in comparison to PsA [6]. Although the ability of REMS to diagnose osteopenia and osteoporosis is well documented in multiple studies, FS has been much less investigated in inflammatory rheumatic diseases. The good concordance between REMS and DXA allows for increased rates of bone density evaluation in clinical practice, with the simultaneous determination of BMD, T-scores, and Z-scores [24]. The risk of femoral fragility fractures is recognized in both axSpA and PsA, although at much lower rates, comparable to those in the general population [33]. The present study, through FS evaluation, demonstrates that there are no significant differences between the two types of conditions, recommending increased vigilance in evaluating any patient presenting with either pathology. 

Study limitations

The lack of determining bone metabolism markers such as osteocalcin and beta crosslaps is a factor that, if included, would have offered a more comprehensive insight into bone modifications in the study context. Furthermore, the short follow-up period only offers a part of the whole picture. The small study groups and the lack of any significant control group may determine difficulties in interpreting the results found (although in the AxSpA group, treatment groups with csDMARDs, bDMARDs, and combined therapies were compared with those without treatment or only taking NSAIDs). Longer studies on larger populations and of different ethnicities and from different geographical regions would provide stronger information regarding bone health under specific medications. An adequate control group will be required in order to fully describe the effect of DMARD therapies on bone health in AxSpA and PsA.

## Conclusions

REMS, through the evaluation of BMD and T-score, as well as through the assessment of the FS, could represent a useful and accurate method for managing patients within the spondyloarthritis group if more studies are performed in this specific context, including different measurement methods. This method provides an overall perspective on bone demineralization and fracture risk, and can be valuable in monitoring these patient groups undergoing various specific therapies. The slight but significant increase in BMD and decrease in FS values, as well as the effect on reducing T-scores, observed both in axSpA and PsA patients, especially under bDMARD therapies and combined csDMARD and bDMARD therapies, may serve as an additional argument for initiating these targeted treatments when clinically indicated, and of course, in accordance with national prescribing protocols. The negative values of 95% CI observed are, most probably secondary to the small study groups and the false-negative values associated with Dunn-Bonferroni testing. Either way, p-values were statistically significant, but further studies are required to fully characterize BMD and FS dynamics in AxSpA and PsA.
